# Modulation of Non‐Rhythmic Temporal Prediction by Subthalamic Nucleus Deep Brain Stimulation (STN‐DBS) in Parkinson's Disease

**DOI:** 10.1002/mds.70165

**Published:** 2026-01-06

**Authors:** Rebecca Burke, Marleen J. Schoenfeld, Christian K.E. Moll, Alessandro Gulberti, Monika Pötter‐Nerger, Andreas K. Engel

**Affiliations:** ^1^ Department of Neurophysiology and Pathophysiology University Medical Center Hamburg‐Eppendorf Hamburg Germany; ^2^ Hamburg Center of Neural and Cognitive Systems University Medical Center Hamburg‐Eppendorf Hamburg Germany; ^3^ Department of Neurology University Medical Center Hamburg‐Eppendorf Hamburg Germany

**Keywords:** cognitive symptoms, deep brain stimulation, Parkinson's disease, temporal prediction task

## Abstract

**Background:**

Accurate temporal prediction, essential for adaptive motor behavior, relies on corticobasal ganglia circuits. In Parkinson's disease (PD), both motor and non‐motor functions are impaired. Deep brain stimulation (DBS) of the subthalamic nucleus (STN) effectively alleviates motor symptoms, but its effects on non‐motor domains, like temporal prediction, remain less understood.

**Objective:**

This study aimed to investigate how STN‐DBS influences temporal prediction performance and its underlying oscillatory dynamics in PD patients, with a particular focus on beta band power and delta band inter‐trial phase consistency (ITPC).

**Methods:**

Thirteen PD patients (5 female, age: 64 ± 5.7 years; disease duration: 11.8 ± 1.8 years) with STN‐DBS performed a temporal prediction task with (DBS ON) and without (DBS OFF) stimulation, while 64‐channel EEG was recorded. Twenty age‐matched healthy controls completed the same task. Behavioral performance was assessed using psychometric functions and time–frequency analyses of electroencephalography (EEG) data on sensor and source‐level examined beta power and delta ITPC.

**Results:**

PD patients showed impaired temporal prediction, but DBS improved performance to a level comparable with controls. EEG revealed reduced beta suppression in PD patients during DBS OFF, while beta suppression in DBS ON was comparable with controls. Both DBS OFF and ON exhibited reduced delta ITPC compared with controls. In DBS ON, source‐level delta ITPC was positively correlated with temporal prediction accuracy.

**Conclusions:**

STN‐DBS improves temporal prediction performance in PD, likely through modulation of beta and delta oscillatory activity. However, while DBS effectively improves behavioral performance, oscillatory mechanisms underlying temporal prediction remain only partially restored. © 2026 The Author(s). *Movement Disorders* published by Wiley Periodicals LLC on behalf of International Parkinson and Movement Disorder Society.

Accurate perception and prediction of time are fundamental for adaptive behavior, enabling humans to anticipate and respond to events in their environment. This ability involves estimating durations in the seconds‐to‐minutes^1^, ^2^ and sub‐second range^3^ and is central to cognitive and motor processes. A key neural substrate underlying interval timing is the basal ganglia (BG), a complex network of structures involved in motor, cognitive, and associative functions. Parkinson's disease (PD), a neurodegenerative disorder characterized by the progressive loss of dopaminergic neurons in the substantia nigra pars compacta, frequently disrupts these functions.^4^, ^5^ The resulting dopamine deficiency slows the pace of the internal clock, leading to impairments in time perception and interval timing tasks.^2^, ^6^, ^7^ Dopaminergic medication has been shown to ameliorate these deficits by modulating the internal clock and enhancing attentional control of temporal information.^8^ However, despite medication, timing performance in PD remains heterogeneous, suggesting the involvement of additional neural mechanisms.^9^ Next to standard dopaminergic medication, deep brain stimulation (DBS) of the subthalamic nucleus (STN) is an established treatment for PD, primarily used to alleviate motor symptoms by reducing excessive beta synchronization within the corticobasal ganglia loops.^4^ Beyond motor improvements, DBS has been shown to have heterogeneous effects on non‐motor functions, including executive processes, working memory, and cognitive flexibility.^10^, ^11^, ^12^, ^13^ Specifically, DBS appears to mitigate PD‐associated impairments in time interval memory retrieval.^14^ By modulating STN activity, DBS provides a unique opportunity to investigate the contribution of the BG in temporal prediction.

In this study, we sought to examine how anticipatory neural dynamics associated with temporal prediction are influenced by DBS in PD. Using electroencephalography (EEG) and a well‐established temporal prediction task,^15^, ^16^ we compared oscillatory markers of anticipation in PD patients once with bilateral therapeutic STN‐DBS turned on (DBS ON), and once with the device switched off (DBS OFF) to those in healthy age‐matched controls. Our findings aim to bridge gaps in understanding the role of the BG in temporal processing and extend previous work on the cognitive effects of DBS.^11^, ^17^ We hypothesized that individuals with PD would show impairments in temporal prediction relative to age‐matched healthy controls, reflected in a shallower slope in psychometric function. Additionally, we hypothesized that these behavioral differences between patients and healthy controls can be linked to altered beta band modulation, in line with previous findings,^18^, ^19^ as well as reduced phase consistency of delta oscillations in the parietal and frontal cortices following stimulus offset.

## 1 Patients and Methods

### 1.1 Participants

Thirteen patients (5 female, mean age: 64 ± 5.7 years) with a diagnosis of advanced idiopathic PD (mean disease duration: 11.8 ± 1.8 years) and 20 healthy age‐matched controls (13 female, mean age: 60.3 ± 3.9 years) took part in the study. The experiment was conducted in accordance with the Declaration of Helsinki and approved by the ethics committee of the Hamburg Medical Association (PV7102). All participants gave written informed consent and received monetary compensation. Handedness was assessed using the short version of the Edinburgh Handedness Inventory.^20^ All participants reported normal or corrected‐to‐normal vision and no history of psychiatric diseases. Participants were excluded if they showed indications of dementia based on a global dementia screening battery (Mini‐Mental State Examination [MMSE] score < 25), psychiatric diseases (per DSM‐IV), neurological conditions other than PD, or were receiving medication for depression. PD patients underwent therapeutical bilateral implantation of DBS electrodes in the STN prior to the experiment (months since surgery: 23.1 ± 15.4) and their daily levodopa‐equivalent daily dose (LEDD), conversion factors as described by Jost et al.^21^ was 786.6 ± 547.4 mg at the time of participation (Table 1). To demonstrate patients' disease progression and severity, we derived the motor‐subscore (Part III) of the Movement Disorder Society‐Unified Parkinson's Disease Rating Scale (MDS‐UPDRS)^22^ and the Hoehn & Yahr (H&Y) scale^23^ from patients' medical history. Controls reported no history of neurological or psychiatric disorders. Both patients (for both sessions in DBS OFF and ON) and controls completed the Trail Making Test A and B,^24^ as well as the digital version of Berg's Card Sorting Test,^25^ to assess their executive functions, specifically cognitive flexibility and logical reasoning. Patients and controls were excluded if they could not perform these tests. Since our focus in the present study was on effects of STN‐DBS on temporal prediction, all patients were on their regular medication for all recordings. While dopaminergic treatment modulates oscillatory dynamics across frequencies,^4^, ^5^, ^26^ both DBS ON and OFF sessions were performed under identical medication conditions, minimizing confounds when comparing stimulation effects. This also ensured patients' physical comfort during the measurement. While the present article focuses on the comparison between PD patients and age‐matched healthy controls, the control data will also be included in a separate publication examining age‐related differences between younger and older adults.

**TABLE 1:** Clinical and demographic characteristics of the Parkinson's disease patients

				Pre‐op UPDRS‐III						Post‐op UPDRS‐III	
Case, gender, age (years)	Disease duration (years)	Pre‐op H&Y	Pre‐op medication (LEDD) (mg)	DOPA‐OFF	DOPA‐ON	DBS device	DBS parameters for: left electrode (1. row), right electrode (2. row)	Post‐op H&Y	Post‐op medication (LEDD) (mg)	DOPA‐OFF DBS‐ON	DOPA‐ON DBS‐ON
1, m, 72	5	2	300	21	12	ME	130 Hz, 1−, 2.0 V, 60 μs 130 Hz, 9−, 2.0 V,60 μs	2	100	26	20
2, m, 65	12	2	1646	42	11	ME	130 Hz, 1−, 2−, 6.0 V, 60 μs 130 Hz, 9−, 5.5 V, 60 μs	2	713	19	13
3, f, 59	12	3	1413	29	13	BS	130 Hz, 2−, 1.5 V, 60 μs 130 Hz, 2−, 1.7 V, 60 μs	1	963	6	5
4, f, 63	11	2	1449	21	13	ME	140 Hz, 1−, 7.5 V, 60 μs 140 Hz, 9−, 6.6 V, 60 μs	2	255	15	10
5, m, 60	10	2	1875	23	17	BS	130 Hz, 2−, 1.1 V, 60 μs 130 Hz, 2−, 2.0 V, 60 μs	2	630	12	11
6, m, 72	15	2	1150	41	17	BS	130 Hz, 1−, 4.3 V, 60 μs 130 Hz, 1−, 5.7 V, 60 μs	2	1253	33	12
7, m, 58	11	2	924	18	9	BS	130 Hz, 2−, 1.8 V, 60 μs 130 Hz, 2−, 1.4 V, 60 μs	1	1862	15	7
8, f, 58	10	2.5	1121	24	9	BS	128 Hz, 1−, 2−, 6.7 V, 60 μs 128 Hz, 2−, 1.4 V, 60 μs	2	350	16	12
9, m, 66	11	1	1442	24	14	BS	130 Hz, 2−, 4.8 V, 80 μs 135 Hz, 2−, 4.5 V, 80 μs	2	1692	17	9
10, f, 71	13	2	1325	35	11	BS	130 Hz, 3−, 2.5 V, 60 μs 130 Hz, 2−, 2.3 V, 60 μs	0	507	25	9
11, m, 70	15	2.5	1181	22	17	BS	130 Hz, 3−, 2.0 V, 60 μs 130 Hz, 3−, 2.3 V, 60 μs	2	971	20	11
12, f, 63	10	3	1000	46	12	BS	130 Hz, 3−, 4.0 V, 60 μs 130 Hz, 2−, 4−, 1.7 V, 60 μs	2	405	34	19
13, m, 59	12	3	1782	31	11	ME	125 Hz, 1−, 5.4 V, 60 μs 125 Hz, 9−, 3.9 V, 60 μs	2	525	9	5

In the column ‘DBS parameters’ values reported are: stimulation frequency in Hertz (Hz), active contacts, impulse amplitude in volts (V), and impulse width in microseconds (μs) for the left and right electrode, respectively. For the left Medtronic (ME) electrode, contact 0 was the most ventral and contact 3 was the most dorsal. For the right ME electrode, contact 8 was the most ventral and contact 11 was the most dorsal. For the left Boston Scientific (BS) electrode, contact 1 was the most ventral and contact 8 was the most dorsal. For the right BS electrode, contact 9 was the most ventral and contact 16 was the most dorsal.

Abbreviations: op, operation for DBS electrodes implantation; UPDRS‐III, Movement Disorder Society‐Unified Parkinson's Disease Rating Scale‐Part III (Motor‐subscore); H&Y, Hoehn and Yahr scale; LEDD, levodopa‐equivalent daily doses; DOPA, levodopa; DBS, deep brain stimulation; M, male; ME, Medtronic; F, female; BS, Boston Scientific.

### 1.2 Experimental Paradigm

**FIG. 1:**
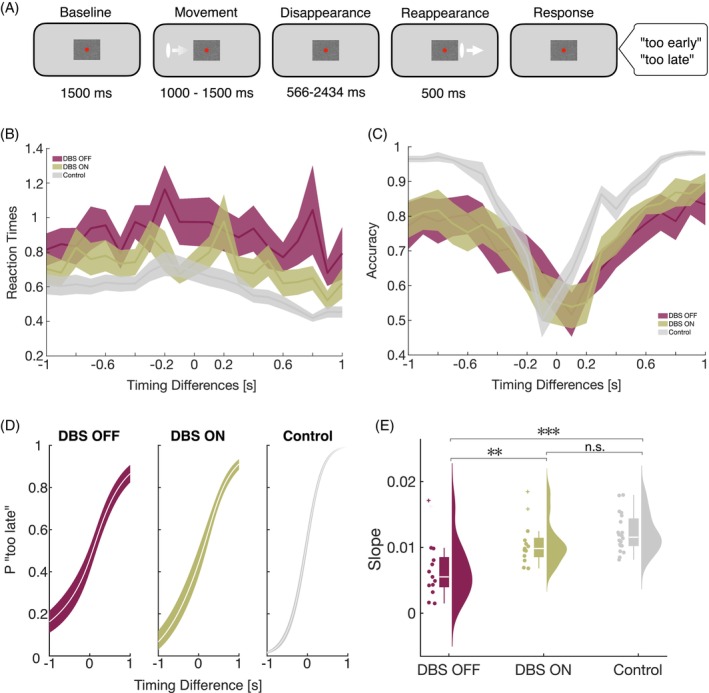
Experimental design and behavioral results. (A) Experimental design of the temporal prediction task is shown. In the beginning of each trial, a white ellipse appeared on the screen and moved towards the occluder before disappearing. After 1500 ms ± 34 to ±934 ms the stimulus reappeared. Participants had to decide whether the stimulus reappeared too early or too late by responding with a keypress. (B) Reaction times and (C) accuracy values for all timing differences are presented as means. (D) Fitted psychometric curves are displayed for patients in the deep brain stimulation (DBS) OFF (red) and DBS ON (green) condition and healthy controls (grey). The timing difference of 0 refers to the objectively correct reappearance of the stimulus after 1500 ms. (E) Slope differences of psychometric functions between conditions and groups are shown as individual data points, boxplots, and distributions. A shallow psychometric curve indicates poorer temporal prediction performance, reflecting lower sensitivity to the timing of events. In contrast, a steep curve reflects better temporal prediction, with more precise anticipation of stimulus timing. A leftward shift of the curve denotes a bias toward predicting events ‘too early’, whereas a rightward shift indicates a bias toward predicting events ‘too late^68^’. Colored/shaded areas depict standard errors of the mean (SEM). *P < 0.05, **P < 0.01, ***P < 0.001; n.s., non‐significant. [Color figure can be viewed at wileyonlinelibrary.com]

The study was conducted in two sessions within 1 week, with consistent timing of day and response mapping. Each session included neuropsychological testing followed by the temporal prediction task (see Fig. 1A; for details on experimental parameters see Daume et al.^15^ and Burke et al.^16^). Participants were seated in a dimly lit, electrically shielded, sound‐attenuated chamber. Visual stimuli were presented on a matte liquid‐crystal display (LCD) screen (1920 × 1080 pixels, 120 Hz refresh rate). Participants fixated a red dot within a white noise occluder throughout the task. Each trial began with 1500 ms fixation, followed by a white ellipse moving left‐to‐right across the screen at constant velocity. The starting position varied, producing movement intervals of 1000–1500 ms. After occlusion, the ellipse reappeared on the right and continued for 500 ms. Reappearance times varied randomly by Δt (±34–934 ms in 100 ms steps) relative to the correct 1500 ms interval. Participants judged reappearance as *too early* or *too late* using a right‐hand button press (mapping counterbalanced). Responses were recorded with a BlackBoxToolKit USB pad.

Controls completed two sessions, interleaved with a more difficult condition (reported separately). Each condition consisted of eight blocks of 60 trials (480 trials/session; 960 total) and the order of conditions was pseudorandomized.

PD patients performed the task as described above in one session with bilateral therapeutic stimulation switched off (DBS OFF) and another session with their stimulation turned on (DBS ON). EEG recordings commenced no earlier than 60 min after the DBS device was turned off. Each session contained 480 trials. All participants completed 30 training trials and received accuracy feedback after each block in the main experiment with a self‐paced block start. Earplugs minimized background noise. Stimuli were presented using MATLAB R2016b (MathWorks, Natick, MA, USA; RRID:SCR_001622) in combination with the Psychophysics Toolbox (RRID:SCR_002881),^27^ running on a Windows 10 operating system.

### 1.3 Data Acquisition and Preprocessing

EEG activity was recorded using a 64‐channel actiCAP snap electrode system (Brain Products GmbH, Gilching, Germany) with active Ag/AgCl electrodes embedded in an elastic cap. Each electrode was equipped with an integrated impedance converter to minimize noise from the environment and movement artifacts, which was particularly crucial for recordings in PD patients experiencing resting tremor. The data were digitized at a sampling rate of 1000 Hz using BrainAmp amplifiers (Brain Products GmbH). Analysis was performed using MATLAB R2019b (The MathWorks Inc., 2019) and two open‐source toolboxes: EEGLAB^28^ (RRID:SCR_007292) and FieldTrip^29^ (RRID:SCR_004849), as well as the MEG and EEG Toolbox Hamburg (METH, Guido Nolte; RRID:SCR_016104) or custom‐made scripts.

### 1.4 EEG Preprocessing/Artefact Removal

Initially, EEGLAB was used to extract data from the experimental blocks. Each of these blocks was filtered with a high‐pass filter at 0.5 Hz to reduce slow drifts, a low‐pass filter at 95 Hz to eliminate high‐frequency DBS artefacts, and a band‐stop filter at 49.5–50.5 Hz for line noise removal. Additionally, the DBSFilt toolbox was employed to remove artefacts in the harmonics and subharmonics of the stimulation frequency. To avoid potential differences in spectral characteristics arising from preprocessing, the DBSFilt toolbox was applied to all datasets (DBS ON, DBS OFF, control). All blocks from each session were subsequently merged and then segmented into epochs of variable lengths using FieldTrip. Each trial was cut from 1240 ms before the stimulus movement onset to 1240 ms after the offset of the reappeared stimulus, resulting in trial durations ranging between 4546 ms and 6914 ms. To remove artifacts from eye movements, muscle activity, and cardiac signals, an independent component analysis (ICA) was performed using the infomax algorithm. Components were identified and rejected based on their time course, variance, power spectrum, and topography through visual inspection. On average, 19 ± 6 of 64 components (DBS OFF: 20 ± 6, DBS ON: 22 ± 9; DBS ON‐DBS OFF: *t*[11]= −1.01, *P* = 0.337, Cohen's *d* = −0.3; Control: 17 ± 4; DBS ON/OFF–Controls: *t*[31] = 2.1, *P* = 0.045, Cohen's *d* = 0.8) were removed per participant per session. After ICA, all trials were visually inspected, and those containing residual artifacts not detected in earlier steps were excluded. On average, 470 ± 5 trials per session remained after preprocessing out of a total of 480 trials.

### 1.5 Quantification and Statistical Analysis

Our primary focus for statistical analyses was to compare data from patients across DBS OFF and DBS ON conditions, as well as to contrast patient data with control group data. To examine differences between DBS OFF and DBS ON conditions within patients, we conducted paired‐samples t‐tests. For comparisons between the patient and control group, we utilized independent‐samples t‐tests. We accounted for multiple comparisons by adjusting the alpha level (α = 0.005/3 = 0.0167).

### 1.6 Behavioral Data Analysis

Participants were not provided with feedback regarding the correctness of their responses. As a result, they made judgments based on their subjective interpretation of what constituted a ‘correct’ reappearance timing. To determine these individual points of subjective equality (PSE), we fitted a psychometric curve to the behavioral data of each participant across all trials in each condition^68^. For each timing difference, we first calculated the proportion of *too late* responses for each participant. A binomial logistic regression (psychometric curve) was then fitted to the data using the *glmfit.m* and *glmval.m* functions in MATLAB. The timing difference corresponding to a 50% *too late* response rate was identified as the PSE for each participant. To evaluate whether there was a significant bias in the PSE values, we compared them to zero using one‐samplet‐tests. The steepness of the psychometric function was quantified as the reciprocal of the difference between the timing differences at 75% and 25% *too late* response rates, providing a measure of the slope of the curve.

### 1.7 EEG Analysis

We decomposed EEG recordings into time–frequency representations using complex Morlet wavelets.^30^ Each trial and channel were convolved with 40 wavelets logarithmically spaced between 0.5 and 100 Hz, with cycles increasing logarithmically from 2 to 10. Only subjectively correct trials (based on individual PSEs; see section on Behavioral Data Analysis) were included. Spectral power was computed across four overlapping windows aligned to task events: baseline (−550 to −50 ms), movement (−50 to 950 ms), disappearance (−350 to 950 ms), and reappearance (−350 to 450 ms), all aligned relative to their respective events. Spectral power estimates were averaged across trials, binned into 100 ms intervals, and normalized using a pre‐movement baseline (−500 to −200 ms). Grand averages across channels were tested against baseline using paired‐sample t‐tests with cluster‐based permutation correction (cluster‐*α* = 0.05, 1000 randomizations). For source‐space analysis, leadfields were computed using a single‐shell volume conductor model^31^ aligned to a Montreal Neurological Institute (MNI) template with a 5003‐voxel grid. Cross‐spectral density (CSD) matrices were derived from wavelet‐convolved data in 100 ms steps and used to compute common adaptive linear spatial filters (DICS beamformer^32^); normalized source power was analyzed using cluster‐based permutation tests to identify significant power differences between groups (two‐sample t‐tests: DBS OFF vs. Controls and DBS ON vs. Controls) and within conditions (paired‐sample t‐tests: DBS OFF vs. DBS ON).

Phase alignment was quantified using inter‐trial phase consistency (ITPC). Phase angles from wavelet‐convolved data were extracted per trial, and ITPC values (0–1) were averaged in 100 ms bins across the same time windows (see earlier). Group and condition comparisons for movement, disappearance, and reappearance windows were tested against baseline using cluster‐based permutation tests. Source‐level ITPC was estimated using the same approach as power analyses, with a focus on delta band activity (0.5–4 Hz) during the disappearance window. Pearson correlations between delta ITPC and psychometric function slope were assessed, again using cluster‐based permutation correction.

## 2 Results

### 2.1 DBS Improved Temporal Prediction Performance

To assess the effects of DBS on temporal prediction performance, we compared the slopes of the psychometric functions between and within groups. Significant differences were observed between conditions of DBS OFF and DBS ON (*t*[12]= −1.68, *P* = 0.007, Cohen's *d* = −1.7), as well as between DBS OFF and Controls (*t*(32) = −5.04, *P* < 0.001, Cohen's *d* = −1.79), but not between DBS ON and Controls (*t*(32) = −1.68, *P* = 0.10, Cohen's *d* = −0.60) (Fig. 1C,D). We found no significant correlation between the slope of the psychometric function and residual motor symptom severity during DBS ON (*r* = 0.108, *P* = 0.752). Because MDS‐UPDRS‐III scores were available only for the DBS ON condition, a direct correlation between DBS‐related changes in temporal prediction and motor symptoms between conditions could not be computed.

### 2.2 DBS Restored Cortical Beta Suppression During Temporal Prediction

**FIG. 2:**
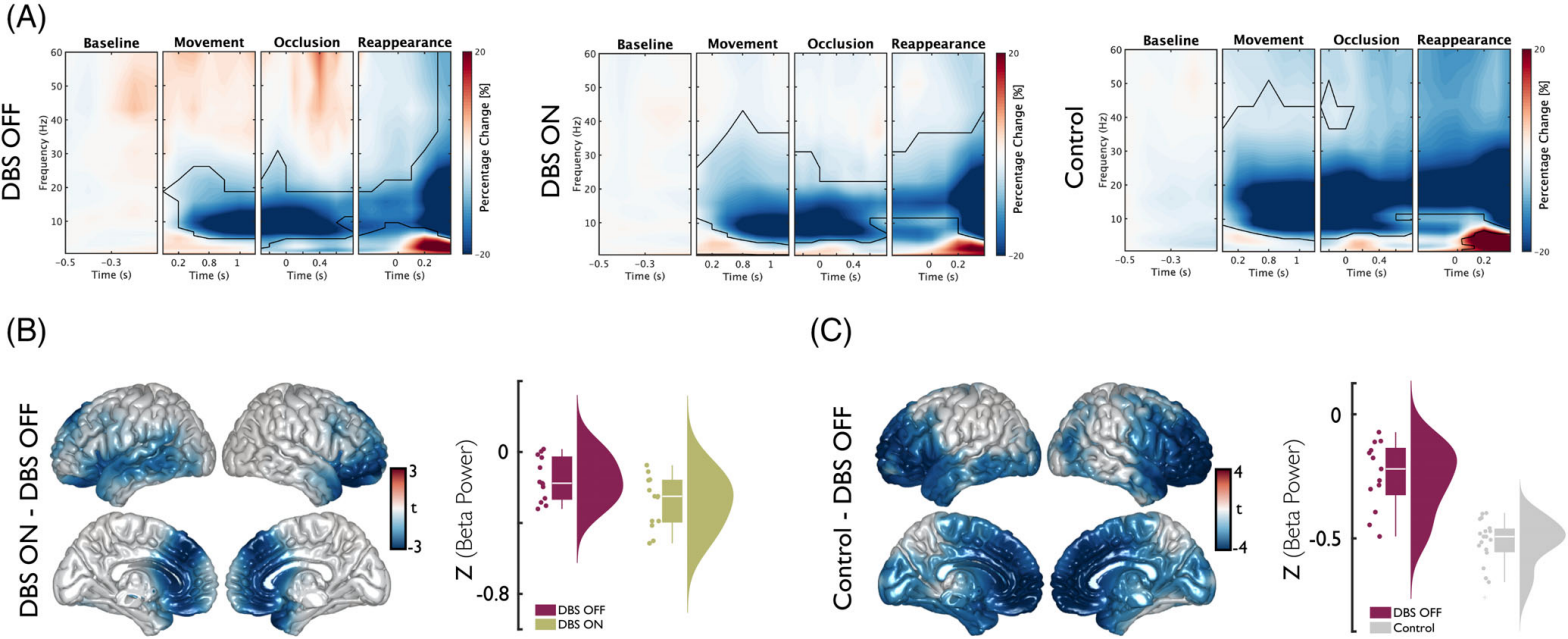
Spectral power modulations across conditions and groups. (A) Spectral power estimates compared with baseline. The panels show time–frequency plots of spectral power, averaged across sensors, conditions, and participants. Each window is centered on their respective event within the paradigm and normalized to the pre‐stimulus baseline. Time 0 s marks the onset of each event. Cluster‐based permutation statistics revealed significant power modulations relative to baseline (indicated by regions enclosed with continuous lines). (B) Source‐level comparison of beta power between deep brain stimulation (DBS) ON and DBS OFF conditions during the disappearance window (−0.2 to 0.6 s). Left: Clusters of voxels with significant differences are highlighted in color. Right: Distribution of z‐scored beta power averaged across voxels showing significant differences between conditions for the time window of disappearance. (C) Left: Source‐level data of beta power differences between Control and DBS OFF for the time window of disappearance (−0.2 s to 0.6 s). Clusters of voxels with significant differences are highlighted in color. Right: Distribution of z‐scored beta power averaged across voxels showing significant differences between groups for the time window of disappearance. [Color figure can be viewed at wileyonlinelibrary.com]

While abnormal alpha‐band (8–12 Hz) oscillations in Parkinson's disease have been linked mainly to non‐motor symptoms, including cognitive and affective dysfunction,^33^, ^34^, ^35^ beta‐band (13–30 Hz) activity has been more closely associated with motor control and temporal processing.^15^, ^18^ In line with this functional distinction, our exploratory analysis did not reveal significant DBS‐related or group differences in alpha activity during the temporal prediction task. In controls, we observed suppression of cortical beta activity during temporal prediction compared with baseline (Fig. 2A). In PD patients, this beta band suppression was generally less pronounced in both DBS OFF and DBS ON conditions compared with controls. Across DBS conditions in patients, beta power appeared to be stronger during DBS OFF than during DBS ON. This observation was supported by the results of cluster‐based permutation statistics, which revealed significant clusters of voxels across bilateral medial prefrontal areas and the left temporal cortex (cluster‐*P* = 0.014; Fig. 2B). Notably, the present analysis reveals a significant decrease in beta power within the medial frontal and left temporal regions in controls when compared with patients with PD during the DBS OFF condition (cluster‐*P* = 0.005; Fig. 2C). However, no significant differences in beta power were observed between the DBS ON condition and controls.

### 2.3 Delta ITPC Was Stronger in Controls Compared with DBS OFF or ON

**FIG. 3:**
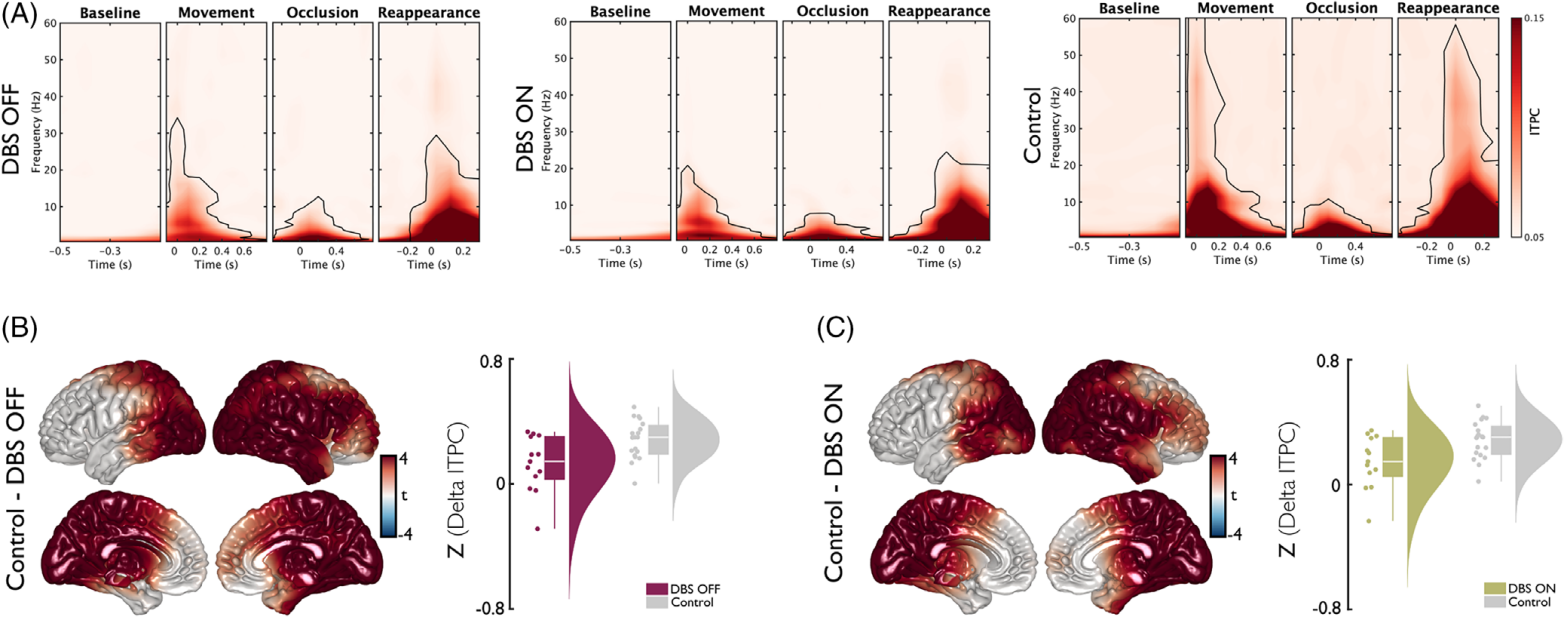
Inter‐trial phase consistency (ITPC) during temporal prediction in patients and controls. (A) The panels show time–frequency plots of ITPC, averaged across sensors, conditions, and participants. Each window is centered on different events within the experiment. Time 0 s marks the onset of each event. Cluster‐based permutation statistics revealed significant ITPC modulations relative to baseline (indicated by regions enclosed with continuous lines). (B) Left: Source‐level data of differences between Control and deep brain stimulation (DBS) OFF within the delta‐band (0.5–4 Hz) for the time window of disappearance (−0.2 to 0.9 s). Clusters of voxels showing significant differences between groups are highlighted in color. Right: Distribution of z‐scored delta ITPC averaged across voxels showing significant differences between groups for the time window of disappearance. (C) Left: Source‐level comparison of ITPC data of Control and DBS ON within the delta band (0.5–4 Hz) for the time window of disappearance (−0.2 to 0.9 s). Clusters of voxels showing significant differences between groups are highlighted in color. Right: Distribution of z‐scored delta ITPC averaged across voxels showing significant differences between groups for the time window of disappearance. [Color figure can be viewed at wileyonlinelibrary.com]

For the ITPC analysis, a similar approach to the one described earlier was used. We found a significant increase in ITPC across various frequency ranges in time bins corresponding to movement onset, occlusion, and stimulus reappearance, respectively (all cluster‐*P* < 0.001; Fig. 3A). The most pronounced ITPC increases for the time window centered around stimulus disappearance, that is, the time window relevant for the temporal prediction process, were found in the delta range in patients as well as controls. Therefore, our group comparisons were focused on frequencies between 0.5 and 4 Hz in a time window of −200 to 900 ms around stimulus disappearance. When comparing delta ITPC of patients with DBS OFF or DBS ON to controls, we identified negative clusters of sensors located in frontal and occipital regions showing significant differences (all cluster‐*P* < 0.001; Fig. 3B). At source level, analysis of the −200 to 900 ms time window revealed the strongest ITPC differences between patients (either DBS OFF or DBS ON condition) and controls in voxels spanning across the occipital, temporal, parietal, and right frontal lobes (all cluster‐*P* < 0.001; Fig. 3C). No significant difference of delta ITPC was found for the comparison of patients with DBS OFF versus DBS ON condition.

### 2.4 Delta ITPC in Patients with DBS ON Was Correlated with Performance

**FIG. 4:**
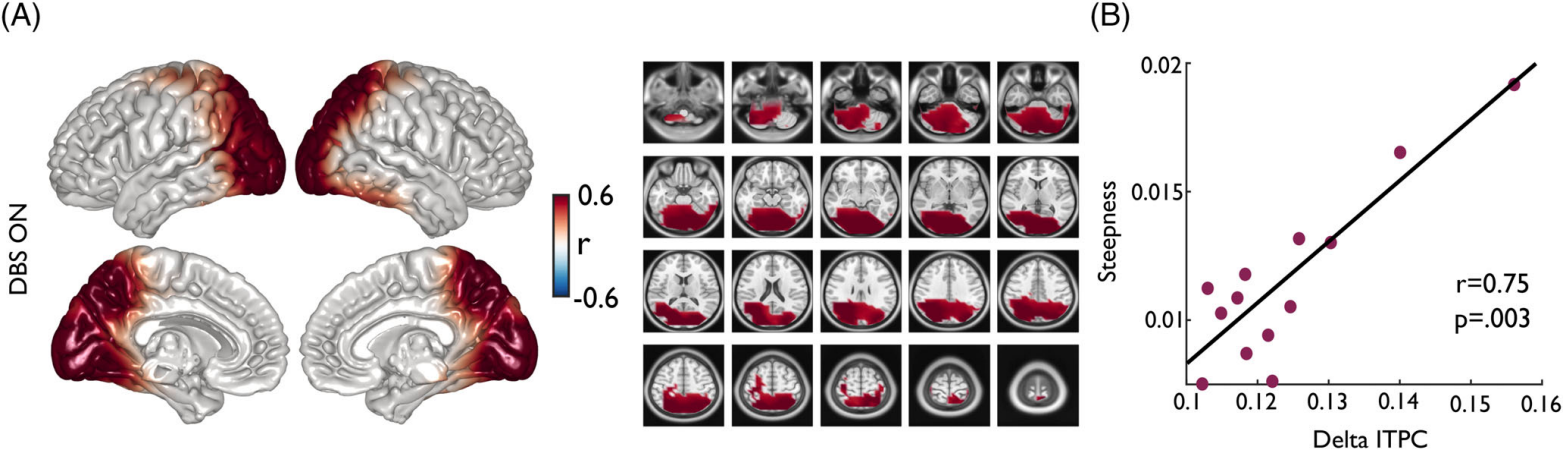
Relationship of delta inter‐trial phase consistency (ITPC) and behavior. (A) Correlation of individual delta ITPC and the slope of the psychometric function within all voxels. ITPC was averaged across the delta band (0.5–4 Hz) and time windows of −0.2 to 0.9 s around the disappearance of the stimulus. Only the clusters of voxels with significant correlations are colored. (B) Each dot of the scatter plot represents one participant. ITPC was averaged across all voxels within the clusters of significant correlations. [Color figure can be viewed at wileyonlinelibrary.com]

To investigate whether the phase alignment of neural oscillations was associated with temporal predictions in patients and controls, we computed Pearson correlations of source‐level delta ITPC with the steepness of the psychometric function. We found a significantly positive correlation only in patients in the DBS ON condition (cluster‐*P* = 0.012; Fig. 4B). Strongest correlations appeared to be in the cerebellum and occipital as well as parietal areas (Fig. 4A). No significant correlation was found for either patients in the DBS OFF condition or controls.

## 3 Discussion

The present study provides novel insights into the modulatory effects of DBS on temporal prediction in PD, offering a new perspective on how BG dysfunction impacts anticipatory cognitive processes. Consistent with our hypothesis and previous studies, PD patients showed impairments in temporal prediction relative to controls, as indicated by a shallower psychometric slope. Importantly, DBS improved performance to levels comparable to controls in the DBS ON condition. Beta suppression was less pronounced in PD patients in the DBS OFF compared with the DBS ON condition and comparable between DBS ON condition and controls. In contrast, delta ITPC was reduced in both DBS ON and OFF conditions compared with controls. For the DBS ON condition, source‐level delta ITPC was positively correlated with temporal prediction accuracy. Our findings underscore that the BG play a significant role in interval timing and highlight that DBS is able to modulate higher‐order cognitive functions.

### 3.1 DBS and Temporal Prediction Performance

Our behavioral results align with earlier studies showing that PD patients exhibit increased variability in timing judgments due to a slowing or disruption of the internal clock mechanism.^2^, ^8^, ^9^ These impairments have been linked to dopamine depletion in the BG, particularly the nigrostriatal pathway, which plays a central role in interval timing and temporal processing.^36^, ^37^ Within this framework, the BG are thought to modulate temporal prediction by integrating sensory and motor information across time, a process that becomes dysfunctional in PD.

The restoration of temporal prediction performance in the DBS ON condition of our study suggests that STN stimulation may partially compensate for disrupted timing mechanisms through the modulation of pathological oscillatory activity and partial normalization of dopaminergic transmission within the cortex‐BG loop.^38^ The observed behavioral improvement of temporal prediction in our study is consistent with prior findings from studies showing that STN‐DBS can enhance time perception and time reproduction.^14^, ^39^ Our results extend these findings by demonstrating that DBS improves not only retrospective time estimation but also predictive timing, which is essential for a wide range of adaptive behaviors, including speech processing, motor coordination, and action planning.^40^, ^41^ Its disruption in PD may contribute not only to motor symptoms like bradykinesia but also to non‐motor deficits in attention, working memory, and executive control.^42^, ^43^ By showing that DBS improves predictive timing, our findings suggest that the therapeutic effects of STN‐DBS extend into the cognitive domain. Notably, stimulation parameters (Table 1) were optimized for motor symptom relief, indicating primary electrode placement in the motor STN. However, the volume of tissue activated likely extended into associative or limbic subregions.^44^, ^45^ Such spread could contribute to cognitive^46^ or neuropsychiatric effects^47^ that may have influenced behavior outcomes. Future investigations combining precise stimulation mapping with behavioral assessment will be critical to elucidate the contribution of each functional subregion to temporal prediction processes.

### 3.2 Beta Activity and the Role of the BG

Beta activity is linked to sensorimotor processing, typically emerging during postural stability and diminishing during active states such as movement preparation and execution.^48^, ^49^, ^50^ Furthermore, it has also been associated with cognitive flexibility and motor readiness.^51^ Consistent with this notion,^15^ we observed pronounced beta suppression in both healthy controls and patients during the temporal prediction task. The reduced beta suppression during DBS OFF compared with patients during DBS ON and healthy controls may reflect the characteristic change in cortical dynamics in PD, where excessive beta activity impairs movement initiation and reduced cognitive adaptability.^5^ Previous research^34^ showed that reduced beta suppression during a working memory task correlated with poorer cognitive performance in PD, suggesting a mechanistic link between beta desynchronization and cognition. In the DBS ON condition, patients showed beta suppression comparable to healthy controls, supporting the idea that STN‐DBS disrupts pathological beta synchronization and restores a more flexible network state.^52^, ^53^ Subcortical–cortical beta coupling is central to this dynamic: beta activity in the STN is tightly coupled to cortical regions via the cortex‐BG loop, and in PD this loop exhibits pathological synchronization and elevated beta cortico‐subthalamic coherence.^5^, ^54^, ^55^, ^56^, ^57^ Both, subthalamic DBS and dopaminergic medication have been shown to attenuate beta power within the STN of PD patients.^50^, ^58^, ^59^, ^60^, ^61^ Thus, when STN‐DBS reduces subcortical beta activity, it may also decouple pathological cortico‐subcortical coherence,^54^ enabling cortical regions, particularly motor and prefrontal areas, to re‐engage in task‐relevant beta desynchronization.^61^, ^62^ Although stimulation is delivered locally to the STN, its broader therapeutic impact likely stems from normalizing beta synchronization across interconnected cortico‐subcortical pathways, thereby enabling greater functional flexibility. Furthermore, our findings may be interpreted in light of the fact that damage to the BG system can lead not only to impaired temporal processing^8^, ^11^ but also to a temporally specific dysfunction in the sequencing of motor actions.^63^ The latter may, in fact, arise as a downstream consequence of disrupted temporal processing, which in turn affects both motor and cognitive domains, resulting in suboptimal timing in perception and action. Within this framework, the observed restoration of beta‐band activity with DBS may reflect improved temporal coordination within these circuits, supporting internally generated timing that underlies both movement execution and higher‐order predictive processes. This interpretation highlights that temporal prediction deficits in PD may not be purely non‐motor phenomena but could rather reflect a shared pathophysiological mechanism linking cognitive and motor timing impairments.

### 3.3 Delta Band ITPC and Temporal Prediction

Beyond power changes, we also examined ITPC in the delta band (0.5–4 Hz), which is associated with the phase alignment of neural oscillations to expected stimuli.^15^, ^64^, ^65^ Both PD patients and healthy controls exhibited increases in delta ITPC during stimulus occlusion and reappearance, consistent with the notion that low‐frequency phase alignment supports the temporal prediction of sensory input. However, both DBS OFF and DBS ON conditions in patients showed reduced delta ITPC compared with healthy controls. Source‐level analyses revealed that the largest discrepancies in delta phase consistency were located in occipital, temporal, parietal, and right frontal cortices, areas involved in attentional and predictive timing.^41^, ^65^ Therefore, while DBS may help recover power dynamics, it may not sufficiently restore the phase consistency necessary for optimal temporal prediction. Interestingly, the correlation between delta ITPC and psychometric function slope was only significant in the DBS ON condition, particularly in the cerebellum, occipital, and parietal regions. These are structures known to support sensorimotor timing and predictive processing.^66^ This indicates that despite reduced ITPC in absolute terms, individual differences in residual phase alignment under DBS ON may still support more accurate temporal judgments. The absence of a similar relationship in DBS OFF and control groups might underscore a potentially unique, compensatory role of DBS in facilitating phase–behavior coupling.

## 4 Conclusions

In summary, this study provides the basic science groundwork for understanding how temporal prediction differs in PD and how it is modulated by DBS. Temporal prediction is essential for coordinated movement, and its disruption may underlie timing‐dependent symptoms such as bradykinesia or freezing of gait. Our study demonstrates that DBS enhances temporal prediction performance in PD by modulating key oscillatory mechanisms, particularly through beta power suppression. This is consistent with a broader body of work suggesting that beta desynchronization facilitates adaptive cognitive processes, including attention and anticipation.^51^ Delta ITPC correlated with temporal prediction performance only in PD patients during the DBS ON condition. Our findings underscore the importance of integrating both power and phase‐based measures when evaluating DBS effects and highlight the potential of oscillatory biomarkers to refine therapeutic strategies for cognitive dysfunction in PD. For instance, ITPC could serve as an additional feedback signal to detect abnormal synchronization during cognitive tasks, allowing adaptive DBS systems to modulate stimulation in a temporally precise, state‐dependent manner. From a translational perspective, optimizing DBS to enhance temporal prediction could, in principle, aid rehabilitation in patients with prominent timing‐dependent symptoms. Virtual reality paradigms that train spatial abilities in PD^67^ might similarly be adapted to target temporal skills. Together, our findings lay the groundwork for future studies linking temporal prediction, motor timing, and DBS‐based rehabilitation in PD.

## Author Roles

(1) Research Project: A. Conceptualization, B. Methodology, C. Investigation, D. Assisted in Patient Recruitment, E. Software, F. Formal Analysis, G. Validation, H. Visualization, I. Data Curation, J. Funding Acquisition, K. Project Administration, L. Supervision; (2) Statistical Analysis: A. Design, B. Execution, C. Review and Critique; (3) Manuscript Preparation: A. Writing of the First Draft, B. Review and Editing.

R.B.: 1A, 1B, 1C, 1D, 1E, 1F, 1G, 1H, 1I,1K, 2A, 2B, 3A, 3B.

M.J.S.: 1A, 1B, 1C, 1D, 1G, 1I, 2C, 3B.

C.K.E.M.: 1C, 1D, 3B.

A.G.: 1C, 1D, 2C, 3B.

M.P.‐N.: 1C, 1D, 3B.

A.K.E.: 1A, 1B, 1J, 1K, 1L, 3A, 3B.

## Financial Disclosures of All Authors (for the Past 12 Months)

The authors declare that the research was conducted in the absence of any commercial or financial relationships that could be construed as a potential conflict of interest. The information and views expressed in this article are purely those of the authors and do not necessarily reflect the official opinion of the European Commission or the European Research Council Executive Agency. R.B., M.J.S., and A.K.E. declare no relevant conflicts of interest. Some of the authors (A.G., C.K.E.M.) have occasionally been reimbursed for travel expenses from Medtronic Inc. C.K.E.M. received lecture and teaching fees from inomed and Abbott. M.P‐N. received lecture or consulting honoraria from Abbott, Boston Scientific, Medtronic, AbbVie, Bial, and Desitin, and received study‐related fees from Boston Scientific, Zambon, and Abbott. The authors declare that there are no additional disclosures to report.

## Financial Disclosures and Conflicts of Interest

Author disclosures are available in the Supporting Information.

## Supporting information


**Data S1.** Supporting Information.

**Data S2.** COI_Disclosure.

## Data Availability

The data that support the findings of this study are available from the corresponding author upon reasonable request.
