# Supplementary material for: Modulation of Non‐Rhythmic Temporal Prediction by Subthalamic Nucleus Deep Brain Stimulation (STN‐DBS) in Parkinson's Disease
Source: Mov Disord. 2026 Jan 6;41(4):992–1003. doi: 10.1002/mds.70165 (PMC13067333; doi:10.1002/mds.70165)
Supplement: Supplementary file 1 — Data S1. Supporting Information. [file MDS-41-992-s001.pdf]

SUPPLEMENTARY MATERIAL

Modulation of non-rhythmic temporal prediction by STN-DBS in Parkinson's disease

Rebecca Burke MSc[1,2], Marleen J. Schoenfeld PhD [1,2], Christian K.E. Moll MD [1], Alessandro Gulberti PhD [3], Monika Pötter-Nerger MD [3], Andreas K. Engel MD [1,2]

[a]Department of Neurophysiology and Pathophysiology, University Medical Center Hamburg-Eppendorf, Martinistr. 52, Hamburg 20246, Germany

[b]Hamburg Center of Neuroscience, University Medical Center Hamburg-Eppendorf, Martinistr. 52, Hamburg 20246, Germany

[c]Department of Neurology, University Medical Center Hamburg-Eppendorf, Martinistr. 52, Hamburg 20246, Germany

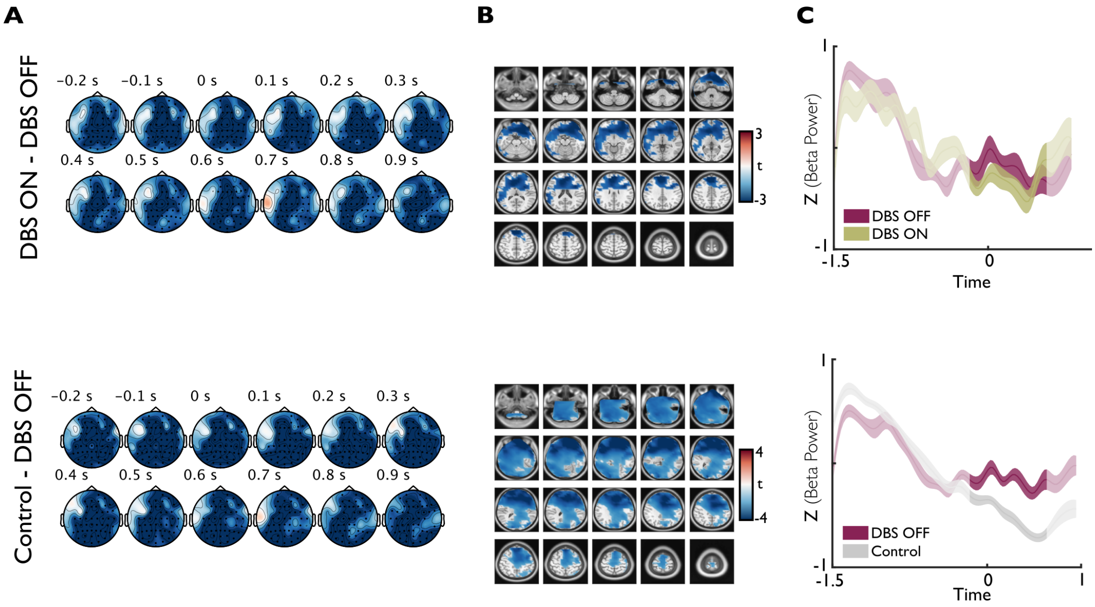

Supplementary Figure 1. Beta power differences between conditions (DBS ON & DBS OFF) and groups (Control & DBS OFF). (A) Sensor level data of beta power differences between DBS ON and DBS OFF (top) and Control and DBS OFF (bottom) for the time window of disappearance (-0.2s to 0.6 s). Clusters of sensors with significant differences indicated by black dots. (B) MRI slices of beta power differences between DBS ON and DBS OFF for the time window of disappearance (-0.2 s to 0.6 s). Clusters of voxels with significant differences are highlighted in color. (C) Time course of beta power (± SEM) averaged across voxels of significant clusters for the difference between conditions and groups. Time 0 s marks the onset of disappearance.

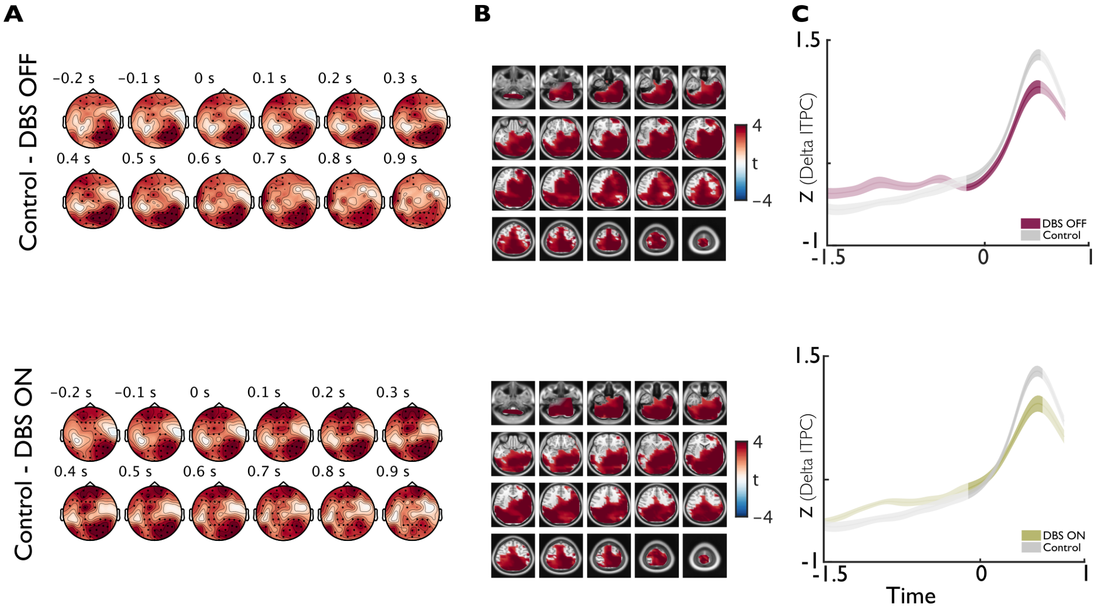

Supplementary Figure 2. Delta ITPC differences between groups. (A) Sensor level data of ITPC differences between Control and DBS OFF (top) and Control and DBS ON (bottom) for the time window of disappearance (-0.2s to 0.9 s). Clusters of sensors with significant differences indicated by black dots. (B) MRI slices of delta ITPC differences between DBS ON and DBS OFF for the time window of disappearance (-0.2 s to 0.9 s). Clusters of voxels with significant differences are highlighted in color. (C) Time course of ITPC (± SEM) averaged across voxels of significant clusters for the difference between groups. Time 0s marks the onset of disappearance.
